# Ensiling as a Conservation Technique for *Opuntia ficus indica* (L.) By-Products: Peel and Pastazzo

**DOI:** 10.3390/ani14223196

**Published:** 2024-11-07

**Authors:** Riccardo Gannuscio, Cinzia Cardamone, Alessandro Vastolo, Caterina Lucia, Angela D’Amico, Giuseppe Maniaci, Massimo Todaro

**Affiliations:** 1 Department of Agricultural, Food and Forest Science (SAAF), Università degli Studi di Palermo, 90128 Palermo, Italy; riccardo.gannuscio@unipa.it (R.G.); caterina.lucia@unipa.it (C.L.); giuseppe.maniaci@unipa.it (G.M.); massimo.todaro@unipa.it (M.T.); 2 Experimental Zooprophylactic Institute of Sicily, 90129 Palermo, Italy; cinzia.cardamone@izssicilia.it; 3 Department of Veterinary Medicine and Animal Production (DMVPA), Università degli Studi di Napoli Federico II, 80137 Naples, Italy; 4 Department of Biological, Chemical and Pharmaceutical Sciences and Technologies (STEBICEF), Università di Palermo, 90123 Palermo, Italy; angela.damico02@unipa.it

**Keywords:** prickly pear by-products, *Opuntia ficus indica* (L.), peels, “pastazzo”, silage

## Abstract

Italy is the third largest producer of Opuntia fruits in the world after Mexico and the United States, and 97.72% of these fruits produced by Italy are grown in Sicily. The use of prickly pear fruits or juice leads to a high production of by-products. In this study, ensiling was chosen to preserve prickly pear peels (PPPs) and “pastazzo” (PPS) mixed with 12% wheat bran. PPP silage presented a lower DM than PPS silage (20.03 vs. 41.37%; *p* < 0.01), as well as aNDFom (25.31 vs. 66.66% DM; *p* < 0.01), but had the best protein content (12.02 vs. 9.55% DM; *p* < 0.01). For both by-products, fermentation proceeded rapidly with increasing temperature, with the temperature for PPS (38 °C) being higher than that for the PPP (30 °C). Mesophilic LAB (lactic acid bacteria) were detected at higher levels than thermophilic LAB, and rod counts were higher than cocci counts. The detected organic acids and silage pH indicate an optimal fermentation process for these by-products. PPP silage had a higher polyphenol content than PPS silage (30.24 vs. 24.22 mg GAE/g DM; *p* < 0.01) and, consequently, also higher antioxidant activity. The results of this study on the mineral composition and macro- and micro-nutrients in silage highlight that these by-products are sources of minerals, with high levels of potassium, calcium, and magnesium.

## Simple Summary

Sustainability is a hotly debated topic, and the livestock sector is considered one of the most important contributors to global warming. The use of by-products as animal feed is beneficial both socioeconomically and ecologically. Prickly pear (*Opuntia ficus-indica* L. Mill) is widely grown in the arid regions of multiple continents, and its by-products can be used as ruminant feed, but their seasonality and chemical properties pose several problems for their preservation and utilization. Ensiling could be the optimal preservation technology. In this study, the chemical composition, nutritional properties, polyphenols, antioxidant activity, and mineral content of prickly pear by-products were evaluated for their persistence after ensiling. It was found that prickly pear by-products are a low-cost and suitable raw material for ensiling; peel silage and “pastazzo” (peel, pulp, and seeds) silage exhibits optimal pH and organic acid content, high polyphenol content, and antioxidant activity and appears to be a source of minerals.

## 1. Introduction

Agro-industrial by-products (AIBPs) are waste products from crop and vegetable processing industries, and their disposal poses environmental problems because they are potential pollutants [1]. Globally, the current disposal and mismanagement of food AIBPs have negative impacts on the environment and social and economic sectors [2,3]. The use of AIBPs as a ruminant feedstock provides a cost-effective solution to valorize residues from agricultural activities, reduce feed costs for livestock farmers, and add value to dairy products locally in a sustainable manner, in accordance with the principles of a circular economy [1,2,3].

Among the AIBPs used to feed ruminants in Sicily, prickly pear (*Opuntia ficus indica* L. Mill.) by-products are an interesting resource [4,5,6,7,8]. These come from the Opuntia cactus, a versatile plant that is widely grown in the arid regions of all the continents for its nutritious fruit and various commercial uses. Italy is the world’s third largest producer of Opuntia fruit, after Mexico and the United States. Sicily is the most productive region of prickly pear fruit in Italy (97.72%), with 8512 hectares of land producing 155,641 tons/year of fresh fruit [9]. The fruit of the prickly pear cactus is consumed mainly as fresh fruit with the peel removed, but the juice may also be extracted for food and medicinal purposes, against diseases such as coronary heart, diabetes, cancers, high blood pressure, inflammation, among others [10]. As a result, large quantities of this AIBP, which is a source of digestible fiber and is rich in bioactive compounds, can be used for animal feeding [8,11].

Prickly pear by-products are obtained after the fruit is peeled for fresh consumption or from the residue of the whole fruit after it is crushed to extract the juice; they consist of the peel, pulp, and seeds, referred to as “pastazzo” [5]. In Sicily, prickly pear by-products become available after mid-August, when fresh forage resources are practically non-existent, and their properties could represent a source of water and digestible fiber for ruminants [12]. As occurs with many AIBPs, seasonality is also a problem for prickly pear by-products; in addition, these by-products need to be preserved before they can be used as feed as their high moisture and sugar content promotes microbial spoilage, the oxidation of organic polymers, and the degradation of bioactive compounds [13]. For long-term use of AIBPs as a livestock feed and throughout the year, it is important to employ simple, low-cost storage methods such as dehydration and/or ensiling [14].

In previous studies, the prickly pear peel (PPP) obtained after peeling the fruits [7] and prickly pear “pastazzo” (PPS) obtained from the residue of whole fruits ground for the extraction of juice [6] were ensiled under laboratory-scale conditions (in vacuum micro-silos). Both silages were made by ensiling the by-products of prickly pear together with other by-products (wheat straw or wheat bran) to increase the dry matter content, which was very low in these raw materials: 8.55% for PPP and 27.68% for PPS. Despite the low dry matter content, the quality level of the silages obtained made ensiling a suitable preservation technology for prickly pear by-products. Gannuscio et al. (2024) [7] reported that silage with the addition of wheat bran is certainly more expensive from an economic point of view compared to silage with the addition of straw, but it was able to reach higher quality levels, which were more pronounced with the addition of 12% wheat bran.

Like other by-products, those of prickly pear are rich in bioactive substances such as polyphenols, proteins, vitamins, and minerals and have multiple physiological activities such as antioxidant, anti-inflammatory, and antibacterial effects [15], which make them suitable for ruminant nutrition.

The aim of this research was to evaluate at a farm level whether the microbiological and nutritional characteristics of prickly pear by-products remain incorporated in silages produced at the farm level.

## 2. Materials and Methods

### 2.1. Ensiling Process

In September 2023, 600 kg of prickly pear peels (PPPs) mixed with 12% bran by raw weight and 600 kg of prickly pear “pastazzo” (PPS) mixed with 12% bran by raw weight were ensiled separately for 50 days in hermetically sealed plastic containers equipped with a degassing valve. The PPP was collected immediately after the fruit was peeled, using an automatic dyeing machine (Agrimat s.r.l.) that separated the skin and the seeds, pulp, and juice. The peel was then transported to a sheep farm where it was immediately ensiled with wheat bran. The PPS was supplied by a produce juice extraction company (Agres s.r.l., Carini, PA, Italy). The fruit was pressed whole in press, the juice was separated, and the remaining “pastazzo” (pulp, peels, and seeds) was loaded onto trucks and, after 24 h, sent to a livestock farm where it was immediately ensiled by adding wheat bran. While filling the silo, dataloggers were inserted inside the ensiled mass with the purpose of detecting the temperature every 30 min until the 50th day.

### 2.2. Physicochemical Analyses

Samples of the PPP, PPS, and wheat bran were taken at the beginning of ensiling, and 57, 70, and 91 days after the closure of the silos, PPP silage and PPS silage samples were taken and transferred to the University of Palermo, at the laboratory of the Department of Agricultural, Food and Forest Science (SAAF). The pH, detected with a pH meter (Hanna Instrument HI3220, Fisherscientific, Oslo, Norway), and water activity, detected at 25 °C using an activity-meter instrument (HC2-AW-USB, Rotronic Int., New York, NY, USA) of the fresh samples were determined. The samples were collected and stored in airtight plastic bags and immediately frozen at −20 °C, and according to the different analyses some of these were freeze-dried. (SCAN-VAC Coolfase 55-9, Labogene Aps, Lynge, Denmark) for successive analysis. The freeze-dried feed samples were then analyzed following the guidelines of the Association of Official Agricultural Chemists (AOAC) to evaluate the levels of dry matter (DM, 934.01), ether extract (EE, 920.39), crude protein (CP, 2001.11), and ash (942.05). For the determination of fiber fractions, the methods proposed by AOAC [16] and Van Soest et al. [17] were used. Specifically, neutral detergent fiber on an organic matter basis (aND-Fom, 2002.04), acid detergent fiber on an organic matter basis (ADFom, 973.18), and acid detergent lignin (973.18) were determined, reporting results excluding residual ash.

### 2.3. Water-Soluble Carbohydrates

The samples of raw materials at the beginning of ensiling and the silage samples 57, 70, and 91 days after the closure of the silos were taken and transferred to the University of Palermo, at the laboratory of the Department of Agricultural, Food and Forest Science (SAAF). The content of the water-soluble carbohydrates (WSC) was measured using a modified anthrone method [18]. Specifically, 0.2 g of freeze-dried and ground samples were sieved through a 1 mm mesh. The samples were then placed in screw-cap Pyrex tubes with 10 mL of water and incubated at 100 °C for 30 min. After cooling, they were centrifuged at 5000 rpm for 10 min, filtered using Whatman 4 filter paper into a 100 mL volumetric flask, and diluted with water. A 1 mL portion of each diluted sample was then transferred to another screw-cap Pyrex tube. Five mL of anthrone solution (0.2% anthrone in concentrated sulfuric acid) was added, and the mixture was heated at 105 °C for 20 min. After cooling in the dark for 30 min, the absorbance at 625 nm was measured using a DR 3900 spectrophotometer (Hach-Lange, Milan, Italy). The content of the water-soluble carbohydrates was calculated according to the calibration curve (R^2^ 0.9979) obtained with glucose standards.

### 2.4. Microbiological Analyses

The samples of the raw materials at the beginning of ensiling and the silage samples 20, 46, 57, 70, and 91 days after the closure of the silos were taken under sterile conditions, kept at 5 °C, and transferred to the microbiological laboratory of the Experimental Zooprophylactic Institute of Sicily.

For each sample, 30 g was diluted 1/10 (*w*/*v*) in 270 mL of buffered peptone water (BPW) and homogenized by a stomacher (Type 400; Seward, London, UK). One mL of each initial suspension and subsequent decimal dilutions were plated for the enumeration of the different microorganisms according to standard methods: total mesophilic microorganism by 4833-1 [19]; *Enterobacteriaceae* by ISO 21528-2 [20]; β-glucuronidase-positive *Escherichia coli* by UNI ISO 16649-2 [21]; coagulase-positive *Staphylococci* by UNI ISO 6888-2 [22]; sulfite-reducing *Clostridium* spp. ISO 15213-1 [23], and yeasts and molds by ISO 21527-1 [24]. Thermophilic and mesophilic rod lactic acid bacteria (LAB) were cultured on de Man–Rogosa–Sharpe (MRS) agar and incubated anaerobically for 48 h at 44 °C and 30 °C, respectively; thermophilic and mesophilic coccus LAB were cultured on Medium 17 (M17) agar containing 5 g/L lactose and incubated anaerobically for 48 h at 44 °C and at 30 °C, respectively.

*Salmonella* spp. and *L. monocytogenes* were detected on 25 g of each sample as described by Todaro et al. (2020) [5].

### 2.5. Organic Acids

Silage samples taken 57, 70, and 91 days after the closure of the silos and subsequently stored at −20 °C were transferred to the University of Naples, Department of Veterinary Medicine and Animal Production (DMVPA), for the determination of organic acids. The samples were analyzed as indicated by Martilotti e Puppo [25]. After thawing, 100 g of each of the silage samples were homogenized with 100 mL of 0.1 N sulphuric acid (insert company). The suspension was quantitatively transferred via powder funnel into a 500 mL volumetric flask, made up to volume with 0.1 N H_2_SO_4_ and shaken. Subsequently, the samples were centrifuged at 15,000 rpm (Universal 32R centrifuge, Hettich FurnTech Division DIY, Melle-Neuenkirchen, Germany). The supernatant of each sample was analyzed in high-performance liquid chromatography (HPLC) to assess the organic acids. The silage extracts were centrifuged at 8000 g for 10 min (Universal 32R centrifuge, Hettich FurnTech Division DIY, Melle-Neuenkirchen, Germany) and filtered; the supernatant (5 µL) was injected in the high-liquid chromatography (Jasco, LC-4000 Series HPLC, 28,600 Mary’s Court, Easton, MD, USA) equipped with a polystyrene divinylbenzene column (Biorad, aminex 85 HPX-87 H, 85,300 × 7.8 mm, 220 nm, flow rate: 0.6 mL/min, T 40 °C) and with a photodiode array detector, using an external standard solution composed of lactic (4.50 mg/L), acetic (5.40 mg/L), propionic (5.76 mg/mL), and butyric (7.02 mg/mL) acids, with 0.008 N sulphuric acid as the mobile phase.

Buffering capacity (BC) was determined according to Martillotti and Puppo [25]. Briefly, 10 g of each silage sample was suspended in 90 g of distilled water and stirred continuously with a magnetic stir bar. The pH of the silage was titrated to pH 3.0 with hydrochloric acid (0.1 N, Fisher Scientific Italia, c/o Segreen Business Park, via San Bovio 3, Segrate (MI), Italia) to eliminate bicarbonates, and subsequently titrated with sodium hydroxide (0.1 N, Fisher Scientific Italia, c/o Segreen Business Park, Segrate (MI), Italia) to a pH of 4.0. Therefore, the titratable alkalinity was expressed in milliequivalents of base required (1 mL 0.1 N NaOH corresponds to 0.1 meq NaOH) to raise the pH of the sample from 4 to 6. Thus, BC was expressed as the amount of base required to produce a change from 4 to 6 in the pH of a 100 g DM silage sample.

### 2.6. Total Phenolic Content and Antioxidant Capacity

The samples of the raw materials at the beginning of ensiling and the silage samples 57, 70, and 91 days after the closure of the silos were taken, stored at −20 °C, and transferred to the University of Palermo, Department of Biological, Chemical and Pharmaceutical Sciences and Technologies (STEBICEF) laboratory. After freeze-drying, 1 g of each feed sample was mixed with 8 mL of methanol (100%) from Sigma-Aldrich (Sigma-Aldrich, St. Louis, MO, USA); the mixtures were vortexed and sonicated in an ultrasonic bath for 40 min. The supernatants were filtered through Whatman 0.45 μm of polytetrafluoroethylene (PTFE) filters. The total phenolic content (TPC) was determined using the optimized Folin–Ciocâlteu method [26], with slight modifications. In brief, an aliquot of each extract (0.125 mL), 120 μL of a 7% Na_2_CO_3_ solution, and 625 μL of Folin–Ciocâlteu reagent (1:5) (Sigma-Aldrich, St. Louis, MO, USA) were incubated in the dark at 25 °C for 60 min. After the incubation, the intensity of color was proportional to the phenolic compound concentration in the sample. The absorbance was evaluated at 765 nm using a UV/Vis spectrophotometer (VWR^®^ UV-1600PC, Milan, Italy). The total phenolic content was calculated based on a calibration curve generated with gallic acid standard solutions (ranging from 0.01 to 0.5 mg/mL). The results were expressed as mg gallic acid equivalents per g of dry matter (mg GAE/g DM).

DPPH and ABTS assays were employed to assess the antiradical activity of the samples. The procedure followed the method previously described by Di Stefano et al. [27]. The DPPH and ABTS tests were used to evaluate the antioxidant activity of a sample through its reaction with solutions of DPPH (2,2 diphenyl-1-picrylhydrazyl) (Sig-ma-Aldrich, St. Louis, MO, USA) and ABTS (2,2′-azino-bis (3-ethylbenzothiazoline-6-sulphonic acid)) (Fluka, Buchs, Switzerland). The effect was a decrease in the color of the solution, proportional to the antioxidants in the sample. For the DPPH assay, the filtrate solutions previously described above for TPC analysis (100 μL) were mixed with 3 mL of DPPH (60 μM) and incubated in the dark at 25 °C for 30 min. The scavenging activity was measured via spectrophotometric analysis of the absorbance at a wavelength of 517 nm with a UV-VIS spectrophotometer (UV-1600PC, VWR). For the ABTS assay, an aliquot of filtered samples (100 μL) was mixed with 3 mL of ABTS, and, after 5 min, the absorbance of the mixture was reading at 734 nm. Methanol (100%) was used as the blank for both assays. Two calibrations curves, using Trolox as the standard at increasing concentrations (1–75 μM), were constructed. The obtained results were reported as mmol Trolox equivalent antioxidant activity (TEAC) and expressed as mmol Trolox equivalent (TEAC) per 100 g of DM (mmol TEAC/100 g DM).

### 2.7. Minerals

The samples of the raw materials at the beginning of ensiling and the silage samples 57, 70, and 91 days after the closure of the silos were taken, stored at −20 °C, and transferred to the University of Palermo, Department SAAF chemistry laboratory. After freeze-drying, the feed samples were analyzed to determine the Ca, Mg, K, Na, Fe, Zn, Cu, Pb, Cd, Ni, Cr, and Mn content after mineralization via the acid digestion procedure. Briefly, 0.5 g of each sample was weighed into a porcelain crucible and placed in a muffle furnace at 550 °C for 8 h. Subsequently, acid digestion of the ashes was performed using 2% HNO_3_ (≥69.0%, Honeywell Fluka, Charlotte, NC, USA) at 100 °C on a hotplate for 15 min. The content of the macronutrients and heavy metals was determined by means of Microwave Plasma Atomic Emission Spectroscopy (MP-AES, Agilent 4210 MP-AES, Milan, Italy). Ca, K, Mg, and Na were reported as g kg^−1^ DM, while the micro-elements were reported as mg kg^−1^ DM.

### 2.8. Statistical Analysis

The qualitative parameters of the raw materials and silages after testing the normality and homoscedasticity of the variance were analyzed with a one-way ANOVA model with the effect of feed as a fixed factor. When the effect of the feed resulted in significance (*p* ≤ 0.05), the means were compared using *p*-values adjusted according to the Tukey–Kramer multiple comparison test. The SAS software (version 9.1, SAS Institute Inc., Cary, NC, USA) procedure GLM was utilized.

## 3. Results

### 3.1. Physicochemical Analyses

The chemical composition of the raw materials and silages is reported in Table 1. As regards the raw materials, the PPP presented significantly lower DM values than the PPS (*p* < 0.01). The addition of 12% wheat bran significantly increased (*p* < 0.01) the DM of both silages by 5% for the PPP and by 3% for the PPS. The protein content of prickly pear by-products was also low and significantly lower (*p* < 0.01) than that of wheat bran, so much so that the addition of bran significantly increased (*p* < 0.01) the crude protein content of both silages. Regarding the fiber fractions, the PPP aNDFom value was lower (28%) than that of the PPS; despite the contribution of fiber due to the addition of wheat bran, the aNDFom value of the PPP silage remained rather lower (38%). The ADL content of the PPS was significantly higher (*p* < 0.01) than that of the wheat bran and PPP, and this higher lignin content was also observed in the PPS silage.

The prickly pear peel showed significantly (*p* < 0.01) higher WSC content than the other by-products used for silage production; after the ensiling process, the WSC content was low, and no differences were found between the silages.

The pH detected in the raw materials showed a significant difference (*p* < 0.01) between the PPP and PPS, highlighting lower values in the prickly pear “pastazzo”. The ensiling significantly reduced the pH of the peel, while the pH of the PPS silage remained near 4.

**Figure 1:**
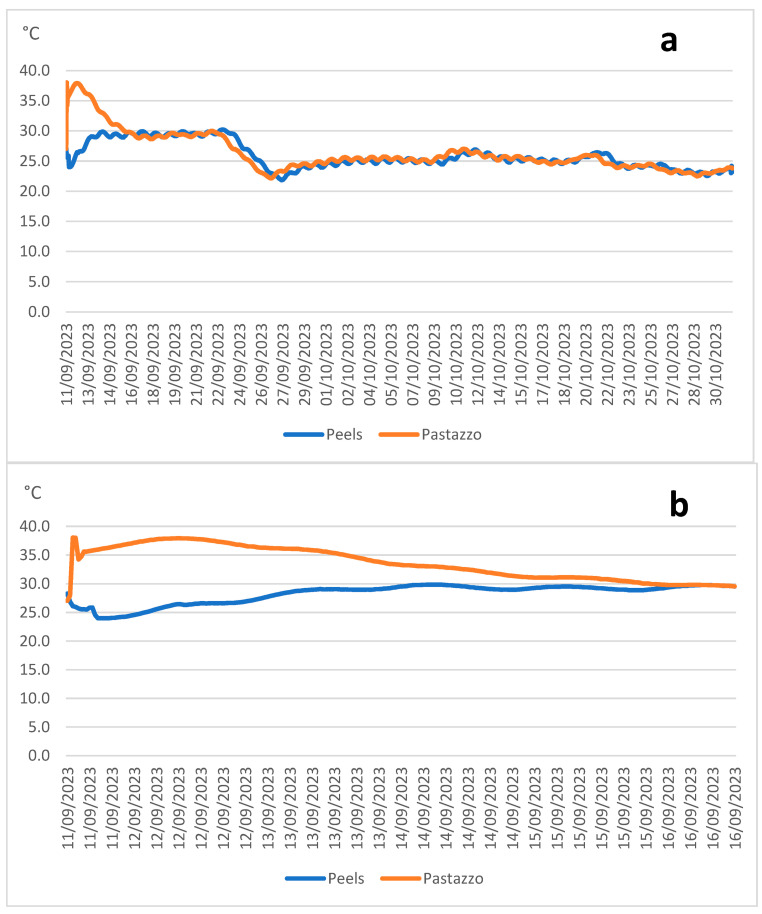
Variation in the temperature of the ensiled by-products ((**a**): first 50 days; (**b**) first 5 days).

Figure 1a shows the results of detecting temperature trends in the silage every 30 min until the opening of the silo, and Figure 1b shows the results of detecting the same trends in the first 5 days, earlier in the fermentation process. From the analysis of these figures, it is clear that the temperature difference displayed by the two silages was high only in the first 5 days of ensiling, and then the temperatures overlapped until the 50th day of ensiling.

Of the two by-products, the PPS reached a higher temperature than the PPP. Both started at 28 °C, but the PPS reached 38 °C after 90 min, remained there for another 24 h, and then slowly began to drop. On the contrary, the temperature of the PPP rose slowly during ensiling, reaching a maximum temperature of 30 °C after about 3 days.

### 3.2. Microbiological Analysis

**Figure 2:**
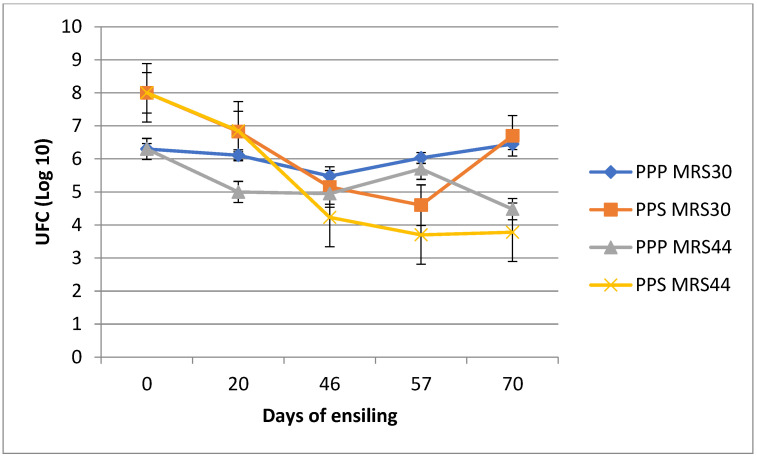
Variation in the mesophilic and thermophilic rod LAB during the ensiling. PPP: prickly pear peel; PPS: prickly pear “pastazzo”; PPP MRS30: mesophilic rod LAB detected in PPP silage; PPS MRS30: mesophilic rod LAB detected in PPS silage; PPP MRS44: thermophilic rod LAB detected in PPP silage; PPS MRS44: thermophilic rod LAB detected in PPS silage.

The trend of lactic acid bacteria (LAB) during the ensiling process was reported in Figure 2. The LAB detected in the PPS and wheat bran mixture showed higher bacteria counts than the PPP mixture before ensiling, and this trend was still observed after 20 days of ensiling.

At 46 days, both of the silages showed the same bacteria counts, but the mesophilic LAB were detected at higher levels than the thermophilic LAB, and this trend was observed until the end of sampling.

The microbiological loads of the raw materials and the silages are shown in Table 2. The raw materials had a lower plate count agar (PCA) than the silages, but only the wheat bran had significantly lower (*p* < 0.01) values than the other materials (58%). The LAB detected in the raw materials showed a more significant presence of bacilli than cocci, while the LAB detected in the silage behaved differently: the bacilli load detected in the raw materials was higher than that in the silage. The thermophile cocci were more abundant in the silage, while they were not detected in the raw material; the load of mesophilic cocci was below 10 CFU/g.

Enterobacteriaceae were detected in wheat bran at three log points and in the PPP at six log points, while the load of VRBGA was below the quantitative levels in the PPS and both of the silages. Mold was detected in the wheat bran and PPP at significantly higher (*p* < 0.01) levels than in the PPS and silage. Yeasts were detected in both of the prickly pear by-products, the PPP and PPS, but after the ensiling process, they were detected only in the PPP silages.

Other spoilage microorganisms, such as coliforms on violet-red bile agar, *Escherichia coli*, coagulase-positive staphylococci, and sulfite-reducing clostridia, were below the detection limits and therefore were not detected in the feed samples. No pathogenic microorganisms, such as *Listeria monocytogenes* or *Salmonella* spp., were detected either in the raw materials or in the silages.

### 3.3. Organic Acids

The organic acids in the silages are reported in Table 3. The lactate level detected in the PPP silage was 15 times higher than that in the PPS silage, while acetate was detected at the same concentration. Therefore, the lactate/acetate ratio of the PPP silage was significantly higher than in the PPS silage. Other differences between the silages were found in the concentration of butyrate, which was higher in the PPP silage than in the PPS silage, and in the buffering capacity, which was significantly higher for the PPP silage than in the PPS silage.

### 3.4. Total Phenolic Content and Antioxidant Capacity

The total phenolic content (TPC) and antioxidant capacity (AOC) are reported in Table 4. In the raw materials, the TPC was significantly higher in prickly pear by-products than in wheat bran, while the TPC detected in the PPP was significantly higher than in the PPS. This trend was also observed in silage; in fact, the PPP silage showed higher TPC values than the PPS silage.

The AOC determined by the DPPH and ABTS assays provided the same results: the PPP presented a higher AOC than PPS. During ensiling, the total phenolic content increased for both the PPP and PPS silages; furthermore, the content in the PPP silage was always higher than in the PPS silage. Both of the tests of the AOC carried out were in accordance with the TPC trend, highlighting how the PPP has a higher AOC than PPS.

### 3.5. Minerals

The macro- and micro-elements in the feeds are reported in Table 5. Both the PPP and PPS were found to be important feeds providing substantial amounts of potassium, calcium, and magnesium. The PPP showed higher values of potassium and magnesium than the PPS, while calcium was more prevalent in the PPS. After ensiling, the levels of potassium and magnesium remained significantly higher (*p* < 0.01) in the PPP silage than in the PPS silage. Regarding these three macro-elements, the wheat bran presented the lowest values. The concentrations of sodium highlighted in all the feeds were modest and did not show significant differences.

Regarding micro-elements, the wheat bran was found to be richer in zinc and copper than the prickly pear by-products; while the PPP presented a significant lower content of iron than PPS, no significant differences were found between the silages. Other differences found between the two silages concerned the manganese content, which was significantly higher in the PPS silage. Finally, as regards the undesirable elements, we found low concentrations of lead only in the wheat bran, but its presence was not found in the silages.

## 4. Discussion

### 4.1. Chemical Composition

The prickly pear by-products used in this study, the PPP and PPS, showed similar characteristics to those used in previous studies [6,7]: the PPP by-product was characterized by lower contents of DM, protein, and aNDFom, while the PPS showed significantly higher values. The third co-product, wheat bran, which was used to increase DM and improve the qualitative characteristics of the silage, contained higher levels of protein and digestible fiber fractions.

Therefore, the addition of 12% wheat bran to the prickly pear by-products significantly increased the DM and protein content of both silages. This addition improved the nutritional properties of these feeds, with a greater effect observed for the PPP silage compared to the PPS silage. Analogous results were found in Morocco, where prickly pear fruit scraps were mixed with wheat bran and straw [28].

The PPP silage had a significantly higher protein content than the PPS silage, even with a lower DM content, with an average value of about 12%, according to the results reported by Gannuscio et al. [7]. The prickly pear peel silage displayed lower aNDFom content than the PPS silage and also a lower indigestible fiber fraction (ADL), probably due to the absence of seeds, which represent a very substantial fraction of the prickly pear by-product [5]. Regarding the ash content, its presence in these by-products was relevant, and the PPP silage showed a significantly higher ash content than the PPS silage. Nevertheless, ruminants can tolerate high mineral intake in the diet and are known to increase their water intake to regulate the osmotic balance in their intestinal tract [29].

The pH of the silages presented optimal values remaining below 4.5, considered the threshold value reported in the literature [30]; similar values were found in the prickly pear silages obtained in laboratory conditions [6,7], highlighting the high quality level of the bacterial fermentation. This high level of fermentation quality was likely due to the WSC content of the prickly pear by-product being higher in the PPP than in the PPS, and it was responsible for the lactic fermentations that determined a lower pH in the PPP silage compared to the PPS silage. The WSC content detected in the PPP was lower than that reported for the same feed (29% of DM) in a previous study [7]. This difference could be justified by the studies of Kuti and Galloway [31], who reported that the relative total glucose and fructose content in the prickly pear fruits differed between species and within fruit tissues; moreover, sucrose was the predominant sugar in the peel samples and was present in smaller quantities in the pulp and juice samples.

### 4.2. Fermentation Process and Microbial Loads

Temperature is a factor that affects silage fermentation, silage quality, and microbial diversity/richness [32]. Generally, a medium temperature of 20 to 30 °C is desirable for silage fermentation. The effects of high temperatures (>37 °C) have been studied because they are well known to be detrimental to forage preservation [33]. The trend in the temperatures detected in the heart of the silos showed an increase in the first 5 days of ensiling, considered the active phase of the fermentation of this by-product [28], and this increase was more noticeable for the PPS than for the PPP, with the consequent reaching of higher temperatures. The higher temperatures reached in the PPS at the beginning of the ensiling were probably due to the different natural microbiota present. In silage fermentation, the substrate was not sterilized, so the microbiota that initiated fermentation consisted of the natural microbiota of the feeds. During fermentation, the microbiota changed based on the characteristics of the feed and the ensiling technique used, progressively changing the genera and species present as the environmental conditions change [34]. The 10 °C difference displayed by the biomass could influence the fermentation process and, consequently, the microbial composition and the quality characteristics of the silage.

Regarding the microbiological characteristics during the ensiling process, Figure 2 reports the trend displayed by mesophilic and thermophilic LAB. As shown in this figure, the PPS LAB count was two log points higher than that of the PPP, likely because the PPS arrived at the farm 24 h after acquisition and it is presumable that the fermentation process had already begun, and this fact was confirmed by the low pH of the PPS raw material. After 20 days of ensiling, the level of lactic bacteria detected in both of the silages decreased, due to the end of the intense fermentation process, as also observed by the trend in the temperatures recorded within the ensiled mass (Figure 1). The four main phases of the fermentation process are the initial aerobic phase, the intense fermentation phase, the stable phase, and the aerobic feed-out phase; the intense fermentation phase lasts several days to several weeks after the silage becomes anaerobic [35]. After 20 days of ensiling, the thermophilic LAB in both the PPP and PPS silages were found at lower counts than the mesophilic ones, probably due to the stabilization of the silage temperatures around 28 °C.

As regards the average load of LAB, we observed the presence of LAB cocci only in the PPP and PPS silages and not in the raw materials; moreover, their counts were lower than those of the LAB rods. LAB populations and epiphytic diversity are highly variable and inhibit the fermentation process. Generally, lactic acid fermentation is initiated when LAB predominate; however, it depends on the proportion of the homofermenting or heterofermenting bacteria, which, in turn, is highly dependent on the availability of substrates and the growth conditions of the predominant microorganisms [36]. The greater presence of lactobacillus found in the prickly pear silages was consistent with Xiang et al. [37], who found that firmicutes and lactobacillus were the dominant taxa during vegetable waste silage fermentation.

Enterobacteriaceae on violet-red bile glucose agar and mold were detected in the raw materials before ensiling but not in the silages. In silage fermentation, the bacteria that initiate fermentation come from the natural microbiota of the feed, but during fermentation, the microbiota changes as the environmental conditions change, and the genera and species present change according to the characteristics of the feed and the ensiling technique used; therefore, the ensiling process does not permit the survival of the VRBGA [34].

Yeasts were detected in the PPP and PPS by-products at five or more log points, but after the ensiling process, they were detected only in the PPP silage at six or more log points. This fact is probably due to the higher sugar concentrations in the PPP, which led to intense yeast growth [33] and to the lower temperature achieved in the PPP silage during the first days of ensiling. In fact, Soundharrajan et al. [36] showed that the fastest yeast growth in corn and wheat silages was found at 20 and 30 °C, while at 40 °C, their activity was reduced. Yeasts are widely distributed in the environment and survive under a variety of temperatures, pHs, and osmotic pressure conditions. Acid tolerance is an undesirable characteristic for silage spoilage microorganisms [34]. The tolerance to acidic environments, the ability to maintain survival in the absence of oxygen, and the ability to use lactic acid as a carbon source allow this microbial population to multiply rapidly [34]. The main problem associated with the presence of yeasts in silage is the high loss of DM in forages containing high concentrations of soluble carbohydrates [38]. Although yeasts are considered undesirable, the term should be used with caution, because some yeasts, mainly Saccharomyces cerevisiae, are widely used as probiotics in ruminants and serve an important function in the rumen [39], and, therefore, further investigations are necessary. However, the microbiological analyses of the raw materials and silage did not reveal any spoilage or pathogenic microorganisms, and this testifies to the high quality of the by-products used and the good ensiling fermentation process obtained.

### 4.3. Organic Acids

The significantly higher presence of lactic acid detected in the PPP silage was probably due to the content of WSC, which was about 15 times higher than in the PPS, and the sugar used by LAB for their fermentation. Furthermore, the higher temperatures reached by the PPS silage could also have made fermentations less homolactic [40]. The high fermentative power of the prickly pear peel has already been highlighted in in vitro fermentation trials by Gannuscio et al. [7], who showed that the lactic acid content detected in similar silages of the prickly pear peel with the addition of 12% wheat bran was 27 g/kg DM. Acetic and propionic acids were detected in the prickly pear silages at low concentrations, and no differences were found between them; furthermore, the concentrations detected in these silages were lower than in other studies on prickly pear silages [6,7]. The concentration of butyric acid detected in the PPP silage was significantly higher than in the PPS silage, but the levels achieved indicate that the silage did not undergo clostridial fermentation, which is one of the poorest fermentations of silages [41].

The percentage of ammonia in the total nitrogen was found to be low for both of the silages, with no significant differences between the silages being found. Similar values were previously found for the PPP silage [6], while values around 15% were reported for the PPS silage [5]. As reported in the literature, well-preserved silages should contain less than 10% of the total N in the form of ammoniacal N [30].

Buffering capacity is the ability of a solution to resist a change in pH through the addition of an acid or an alkali; therefore, the BC of the silage was defined as the ability of a given amount of this feedstuff to resist a change in pH after the addition of either an acidic or a basic solution [42]. The BC was significantly higher in the PPP silage than in the PPS silage, probably because the addition of wheat bran to the prickly pear by-product increased the CP content in the silage more in the PPP silage than in the PPS silage. In fact, wheat bran is rich in protein and amino groups, and these compounds contribute to the increased buffering effect of silage [43,44].

### 4.4. Total Phenolic Content (TPC) and Antioxidant Capacity (AOC)

The TPC detected in prickly pear by-products was threefold higher than in wheat bran. It is well known that prickly pear fruit is richer in secondary metabolites, mainly polyphenols, such as flavonoids, carotenoids, and anthocyanins [44]. The TPC was higher in the PPP than in the PPS, in accordance with what was reported by other authors who found a significantly higher TPC in the prickly pear peel than in prickly pear pulp or seeds [45,46,47,48]. In our study, PPP and PPS silages showed higher TPC values than the respective raw materials, similar to that reported by Łozicki et al. [49] on pumpkin (*Cucurbita maxima* D.) silage. This effect could be explained by the LAB activity that produced a β-glucosidase enzyme, which catalyzed the release of phenols during the ensiling, making them more accessible to the solvent during the extraction [50]. These may have increased the antiradical potential of the silage, due to the relationship between the content of phenolic compounds and the antioxidant potential of plant products [51].

Moreover, the TPC detected in the Sicilian prickly pear peel was definitely higher than those reported for the prickly pear peel cultivated in Tunisia (1.3–2.9 mg GAE/g [44]), Egypt (3.85 mg GAE/g [43], 14.58 mg GAE/g [47]), Mexico (8.62–12.28 mg GAE/g [10], 3.50–3.83 mg GAE/g DM [46], 3.06–14.30 mg GAE/g [45]), and Spain (3.56–4.46 mg GAE/g DM [46]). The differences between the TPC reported for different cultivars and different areas could be caused by environmental factors such as drought stress, soil nutrition, sun exposure, and ambient temperature [45].

The antioxidant capacity (AOC), generally, was related to the chemical composition of the prickly pear by-products, attributed to their richness in total phenolic content [45]. Both analyses of the DPPH and ABTS assays, produced the same results, showing that the AOC in the PPP was higher than in the PPS for both the by-products and the silage. This is probably due to the fact that PPS also contains pulp and seeds, presenting a lower TP concentration than peels. This lower AOC found in the PPS was consistent with the results reported by other authors [45,46,47]. These results confirm that prickly pear peels are the most important source of phytochemical compounds and, for this reason, the AOC of the prickly pear fruit could be taken into consideration in future applications for the use of this by-product.

### 4.5. Minerals

The roles of minerals in animal organisms are very diverse and closely related to their form and condition. The main functions include involvement in the formation of connective tissue, the homeostasis of body fluids, direct and indirect effects on endocrine gland function, and influences on the microbiota (symbiotic microbiota) of the digestive tract [52].

According to other authors, prickly pears are regarded as a rich supplier of the minerals potassium, calcium, and magnesium [44,53,54], and the quantities detected in the PPP and in the PPS silages were more than sufficient to ensure the mineral needs of dairy sheep [55]. Calcium and potassium were the macro-elements most commonly present in our prickly pear silages and the concentrations detected were consistent with the data reported by other authors [44,53,54]. However, it is well-known that the location of the plants, the method of cultivation, the application of fertilizers and irrigation, the climate, and genetic variances can influence the mineral composition of fruits [55]. Magnesium was detected at higher levels in the PPP than in the PPS, and analogous results were found by Arafa et al. [53], who found that the value in the PPP was double that in seeds, and by El Kossori et al. [54], who found higher values in the PPP with respect to seeds and pulp. The presence of magnesium in the diet is necessary for the processes that transform vitamin D into its active form and, as a result, produce adenosine triphosphate (ATP), a component that releases the parathyroid hormone and relaxes the muscles [56]. The nutritional requirements of sheep are already well-established; comparing the mineral levels in the PPP and PPS silages with the requirements and maximum values allowed for dairy sheep and considering a silage supplementation in the diet of maximum 1.5 kg/day/head, the levels of calcium and magnesium were in accordance with the daily requirement [54], while the level of potassium exceeded the daily requirement [54].

Among the micro-elements present, zinc and iron were less prevalent in prickly pear peels and more prevalent in seeds [53,54], meaning that the PPPs presented significantly lower zinc and iron contents than the PPS. In contrast, wheat bran had high zinc contents (approximately six–eight-fold higher) and iron contents compared to the prickly pear by-products; therefore, its use in the silage mix helped to increase the amounts of zinc and iron in the silage. Even though zinc deficiency in ruminants is rare because of the fairly high zinc content of grasses in natural and cultivated pastures, the function of zinc is to maintain a clear arrangement of RNA, which indirectly affects protein biosynthesis and genetic information transfer [51]. Iron is widely encountered in plants and animals, in which it is an essential component, and its contents in plants varies with the species, vegetative stage, soil type, and environmental pollution. Even though the iron requirements of farm animals are usually satisfied by natural feeds, some problems of iron deficiency may occur in suckling animals [51].

Manganese and copper are present in both prickly pear by-products and silages, and these micro-elements are important because they are used for bone mineralization, muscle contraction, nerve stimulus transmission, and act as a cofactor of many enzymes involved in human metabolism [44]. Finally, the concentration of the micro-elements detected in the PPP and PPS silages, especially manganese, copper and zinc, was below the toxic concentration for sheep [52,55]; therefore, no problems could arise due to the mineral intake provided by prickly pear by-products.

## 5. Conclusions

Prickly pear by-products are characterized by low dry matter content, a fact that could be a negative factor for the ensiling process. However, the results found in this study highlight how PPP and PPS silages are suitable low-cost feeds for ruminant nutrition. The silages had an optimal pH and organic acid contents, with high levels of beneficial LAB counts and without the presence of spoilage or pathogenic bacteria. The total content of the polyphenols was found to be high compared to the other prickly pear by-products analyzed in other countries; consequently, the antioxidant capacity was found to be high in both the by-products and the silage, highlighting its persistence during ensiling. Additionally, the minerals, including both macro-elements and micro-elements, highlighted that these by-products are a source of minerals, particularly potassium, calcium, and magnesium.

The differences found between the nutritional characteristics of the PPP and PPS silages highlight better results for the PPP silage, which is also richer in polyphenols and, consequently, has greater antioxidant activity; furthermore, its richness in macro- and micro-elements makes PPP silage a mineral supplement without toxicity problems for sheep.

## Figures and Tables

**Table 1:** Chemical composition of feeds (% DM).

Items	Raw Materials			Silages		SEM	*p*-Value
	Bran	PPP	PPS	PPP	PPS		
Dry matter	89.17 ^A^	14.97 ^E^	38.47 ^C^	20.03 ^D^	41.37 ^B^	0.516	0.001
Crude protein	17.86 ^A^	5.16 ^E^	6.52 ^D^	12.02 ^B^	9.55 ^C^	0.250	0.001
Ether extract	5.07 ^ABb^	1.49 ^CDd^	3.10 ^BDc^	6.15 ^Aa^	3.78 ^Bb^	0.520	0.001
aNDFom	28.96 ^C^	20.45 ^D^	71.89 ^A^	25.31 ^C^	66.66 ^B^	0.734	0.001
ADFom	9.34 ^D^	10.10 ^D^	57.68 ^A^	11.38 ^C^	50.03 ^B^	0.315	0.001
ADL	3.16 ^C^	1.63 ^C^	36.12 ^A^	2.18 ^C^	26.77 ^B^	0.164	0.001
NFC	43.02 ^B^	60.69 ^A^	9.90 ^C^	45.01 ^B^	11.92 ^C^	0.820	0.001
WSC	4.16 ^B^	18.60 ^A^	1.50 ^B^	3.36 ^B^	1.10 ^B^	1.051	0.001
Ash	5.54 ^C^	12.43 ^A^	8.70 ^B^	11.52 ^A^	8.08 ^B^	0.354	0.001
pH	5.07 ^B^	5.71 ^A^	4.15 ^C^	3.75 ^C^	4.04 ^C^	0.144	0.001
Activity water	0.66 ^C^	0.97 ^A^	0.97 ^A^	0.96 ^AB^	0.95 ^B^	0.005	0.001

PPP: prickly pear peel; PPS: prickly pear “pastazzo”; aNDFom: neutral detergent fiber assayed with a heat stable amylase and expressed exclusive of residual ash; ADFom: acid detergent fiber expressed exclusive of residual ash; ADL: acid detergent lignin; WSC: water-soluble carbohydrate; NFC: non-fiber carbohydrate = 100 − (CP + ether extract + ash + aNDFom); SEM: standard error of mean; on the row, the values with different superscript letters are significant A, B, C, D, E: *p* ≤ 0.01; a, b, c, d: *p* ≤ 0.05.

**Table 2:** Microbiological analysis of feeds (Log_10_/g).

Items	Raw Materials			Silages		SEM ^4^	*p*-Value
	Bran	PPP	PPS	PPP	PPS		
Plate Count Agar (PCA)	3.94 ^B^	6.72 ^A^	6.70 ^A^	7.41 ^A^	7.40 ^A^	0.16	0.002
MRS 30 °C ^1^	3.34	8.00	8.00	4.83	5.67	1.09	0.405
MRS 44 °C ^1^	3.23	8.00	8.00	4.10	3.16	0.85	0.117
M17 44 °C ^2^	<1 ^b^	<1 ^b^	<1 ^b^	2.43 ^a^	3.38 ^a^	0.38	0.040
VRBGA ^3^	3.38	6.41	<1	<1	<1	-	-
Mold	2.95 ^A^	3.08 ^A^	2.08 ^B^	1.10 ^B^	<1 ^B^	0.07	0.002
Yeasts	<1 ^B^	5.48 ^Ab^	5.00 ^Ab^	6.30 ^Aa^	<1 ^B^	0.12	0.001

PPP: prickly pear peel; PPS: prickly pear “pastazzo”; ^1^ MRS, de Man–Rogosa–Sharpe agar for rod LAB; ^2^ M17, agar for coccus lactic acid bacteria (LAB); ^3^ violet-red bile glucose agar for Enterobacteriaceae (VRBGA); ^4^ SEM: standard error of mean. On the row, values with different superscript letters are significant; A, B: *p* ≤ 0.01; a, b: *p* ≤ 0.05.

**Table 3:** Fatty acid content of silages.

	PPP Silage	PPS Silage	SEM	*p*-Value
Lactate (g/kg DM)	20.02 ^A^	1.26 ^B^	1.167	0.001
Acetate (g/kg DM)	1.96	1.92	0.145	0.846
Propionate (g/kg DM)	0.482	0.236	0.171	0.334
Butyrate (g/kg DM)	0.699 ^A^	0.020 ^B^	0.054	0.001
Lactate/acetate	10.54 ^A^	0.68 ^B^	0.723	0.001
N-NH_3_/N (g/100 g)	1.277	1.525	0.204	0.411
Buffering capacity (meq NaOH/100 g DM)	114 ^A^	78 ^B^	4.25	0.001

PPP: prickly pear peel; PPS: prickly pear “pastazzo”; SEM: standard error of mean. On the row, values with different superscript letters are significant; A, B: *p* ≤ 0.01.

**Table 4:** Total phenolic content and antioxidant capacity of feeds.

Items	Raw Materials			Silages		SEM ^4^	*p*-Value
	Bran	PPP	PPS	PPP	PPS		
TPC ^1^ (mg GAE/g DM)	8.69 ^E^	28.96 ^B^	20.79 ^D^	30.24 ^A^	24.22 ^C^	0.11	0.001
DPPH ^2^ (mmol TEAC/100 g DM)	4.02 ^E^	11.57 ^B^	7.85 ^D^	15.75 ^A^	10.06 ^C^	0.13	0.001
ABTS ^3^ (mmol TEAC/100 g DM)	12.62 ^E^	25.55 ^B^	17.69 ^D^	26.46 ^A^	21.58 ^C^	0.14	0.003

PPP: prickly pear peel; PPS: prickly pear “pastazzo”; ^1^ TPC: total phenolic content; ^2^ DPPH: 2,2-diphenyl-1-picrylhydrazyl index; ^3^ ABTS: 2,2′-azino-bis 3-ethylbenzothiazolino-6-Sulphonic acid; ^4^ SEM: standard error of mean. On the row, values with different superscript letters are significant; A, B, C, D, E: *p* ≤ 0.01.

**Table 5:** Mineral composition of feeds.

Items	Raw Materials			Silages		SEM ^1^	*p*-Value
	Bran	PPP	PPS	PPP	PPS		
Ca (g kg^−1^ DM)	2.72 ^Bc^	20.78 ^Ab^	31.51 ^Aa^	23.50 ^Aab^	22.48 ^Ab^	2.00	0.001
K (g kg^−1^ DM)	13.40 ^Bc^	40.51 ^Aa^	23.98 ^Bb^	43.43 ^Aa^	20.40 ^Bbc^	3.30	0.001
Mg (g kg^−1^ DM)	3.37 ^C^	11.21 ^A^	5.54 ^B^	10.01 ^A^	5.39 ^B^	0.63	0.001
Na (g kg^−1^ DM)	0.41	0.51	0.46	0.54	0.41	0.17	0.977
Zn (mg kg^−1^ DM)	120.5 ^A^	11.21 ^E^	21.16 ^D^	55.51 ^B^	45.81 ^C^	2.39	0.001
Cd (mg kg^−1^ DM)	nd	nd	nd	nd	nd	-	-
Fe (mg kg^−1^ DM)	229.1 ^Bbc^	33.7 ^C^	174.5 ^Bc^	266.6 ^ABb^	354.7 ^Aa^	23.26	0.001
Cu (mg kg^−1^ DM)	16.26 ^A^	6.68 ^CDd^	5.24 ^D^	12.27 ^B^	8.84 ^Cc^	0.60	0.001
Ni (mg kg^−1^ DM)	0.75 ^D^	0.32 ^E^	1.41 ^B^	1.07 ^C^	2.21 ^A^	0.07	0.001
Pb (mg kg^−1^ DM)	0.60	nd	nd	nd	nd	-	-
Mn (mg kg^−1^ DM)	81.14 ^Bbc^	67.26 ^Bc^	75.09 ^Bbc^	86.42 ^Bb^	113.74 ^Aa^	6.21	0.004
Cr (mg kg^−1^ DM)	3.45 ^A^	3.34 ^A^	2.97 ^A^	1.75 ^B^	1.35 ^B^	0.27	0.001

PPP: prickly pear peel; PPS: prickly pear “pastazzo”; nd: non-detected; ^1^ SEM: standard error of mean. On the row, values with different superscript letters are significant; A, B, C, D, E: *p* ≤ 0.01; a, b, c, d: *p* ≤ 0.05.

## Data Availability

The original contributions presented in this study are included in the article; further inquiries can be directed to the corresponding author/s.
